# Ameliorative effects of omega-lycotoxin-Gsp2671e purified from the spider venom of *Lycosa praegrandis* on memory deficits of glutamate-induced excitotoxicity rat model

**DOI:** 10.3389/fphar.2022.1048563

**Published:** 2022-12-16

**Authors:** Mohammad Keimasi, Kowsar Salehifard, Marzieh Shahidi, Fariba Esmaeili, Noushin Mirshah Jafar Esfahani, Siamak Beheshti, Mohammadreza Amirsadri, Faezeh Naseri, Mohammadjavad Keimasi, Najmeh Ghorbani, Mohammad Reza Mofid, Majid Moradmand

**Affiliations:** ^1^ Department of Plant and Animal Biology, Faculty of Biological Sciences and Technology, University of Isfahan, Isfahan, Iran; ^2^ Department of Physiology, School of Medicine, Isfahan University of Medical Sciences, Isfahan, Iran; ^3^ Department of Clinical Pharmacy and Pharmacy Practice, School of Pharmacy and Pharmaceutical Sciences, Isfahan University of Medical Sciences, Isfahan, Iran; ^4^ Department of Clinical Biochemistry, Faculty of Medicine, Shahid Beheshti University of Medical Sciences, Tehran, Iran; ^5^ Department of Clinical Biochemistry, School of Pharmacy and Pharmaceutical Sciences, Isfahan University of Medical Sciences, Isfahan, Iran

**Keywords:** animal venoms, spider, calcium channel modulators, calcium channel Cav2.1 (P/Q type), memory

## Abstract

Memory impairment is one of the main complications of Alzheimer’s disease (AD). This condition can be induced by hyper-stimulation of N-Methyl-D-aspartate receptors (NMDARs) of glutamate in the hippocampus, which ends up to pyramidal neurons determination. The release of neurotransmitters relies on voltage-gated calcium channels (VGCCs) such as P/Q-types. Omega-lycotoxin-Gsp2671e (OLG1e) is a P/Q-type VGCC modulator with high affinity and selectivity. This bio-active small protein was purified and identified from the *Lycosa praegrandis* venom. The effect of this state-dependent low molecular weight P/Q-type calcium modulator on rats was investigated *via* glutamate-induced excitotoxicity by N-Methyl-D-aspartate. Also, Electrophysiological amplitude of field excitatory postsynaptic potentials (fEPSPs) in the input–output and Long-term potentiation (LTP) curves were recorded in mossy fiber and the amount of synaptophysin (SYN), synaptosomal-associated protein, 25 kDa (SNAP-25), and synaptotagmin 1(SYT1) genes expression were measured using Real-time PCR technique for synaptic quantification. The outcomes of the current study suggest that OLG1e as a P/Q-type VGCC modulator has an ameliorative effect on excitotoxicity-induced memory defects and prevents the impairment of pyramidal neurons in the rat hippocampus.

## Introduction

Alzheimer’s disease (AD) is the most common neurodegenerative disorder (Naseri et al., 2022). It is characterized by progressive memory loss and cognitive deficits. It directly affects the lives of millions of people worldwide (Kumar and Singh, 2015). One of the significant mechanisms underlying the progression of AD is the over-activity of glutamate receptors following the increased function of calcium channels and high glutamate release from the presynaptic neurons in hippocampus (Schurr, 2004; Nimmrich and Gross, 2012). Many studies show that over-activation of glutamate receptors plays a crucial role in developing and progressing the neurodegenerative disorders and neurotoxicity, known as excitotoxicity (Dong et al., 2009; Mehta et al., 2013; Lai et al., 2014). Overstimulation of these receptors in neurotoxicity research models results from the administration of glutamate receptors agonists (Jarrard, 2002; Zhang et al., 2014). N-Methyl-D-aspartate (NMDA) can cause a significant release of glutamate as an excitatory neurotransmitter by affecting the presynaptic P/Q-type voltage-gated calcium channels (VGCCs) (Mochida, 2019). Intra-hippocampal injection of NMDA has been used to induce excitotoxicity, which is very similar to the neurotoxicity in AD. The main manifestations of this toxicity are forgetfulness and memory impairment which are hall markers for AD pathology (Kumar and Singh, 2015).

Typical N-Methyl-D-aspartate receptors (NMDARs) activity is vital for learning and memory performance. Balanced stimulation of glutamate receptors particularly NMDAR, is necessary for learning and memory formation by long-term potentiation (LTP) process (Hayashi, 2022). It plays an essential role in these processes, including increasing dendrites, and synaptic plasticity in the brain (Bin Ibrahim et al., 2022). This can be measured by synaptophysin (SYN), synaptosomal-associated protein, 25 kDa SNAP-25, and synaptotagmin 1(SYT1) (Li and Kavalali, 2017). SYN is a calcium-dependent protein in presynaptic neurons. Also, SYN is the major synaptic vesicle protein in typical state of brain (Rehm et al., 1986). But, its amount diminishes in the AD (Li and Kavalali, 2017). This reduction has a direct link with memory impairment in the AD. This protein is a marker for synaptic performance (Zhang et al., 2014; Ji et al., 2017). SNAP-25 is a part of soluble N-ethylmaleimide-sensitive factor activating protein receptor protein complex, which has a fundamental role in release of neurotransmitters. The presence of SNAP-25 is necessary for long-term potentiation (LTP). Also, the SNAP-25 has an important role in cognitive ability (Zhang et al., 2014; Biswal et al., 2017). SYT1 is a calcium-sensor for fast vesicle exocytosis. Decrease in SYT1 is a marker for synaptic pathology in AD (Zhang et al., 2014; Hussain et al., 2017). Therefore, the measurement of SYN, SNAP-25 and SYT1 genes expression can be used as indexes for synaptic function. On The other hand, excessive stimulation of NMDARs can lead to destructive reactions in neuronal cells, which ends up to pyramidal neurons death in the hippocampus (Arundine and Tymianski, 2003; Szydlowska and Tymianski, 2010). Over-activity of the presynaptic VGCCs (N, and P/Q-type) involved in neurotransmitter release, is one of the causes of this over-stimulation (Nimmrich and Gross, 2012; Sousa et al., 2013; Verma et al., 2022). Meanwhile, the accumulation of calcium in mitochondria ends to the production of various free radical species and activation of the internal pathway of apoptosis (Verma et al., 2022). These events lead to induction of apoptosis in nerve cells and neuronal death, reduction of neurotrophic factors, and decreased plasticity (Szydlowska and Tymianski, 2010).

Venom is a complex biochemical compound that is produced, and stored in living organisms, such as snakes, scorpions, bees, and spiders (Moradi et al., 2018). The use of venom proteins has many advantages, due to their low molecular weight, high solubility, and stability (because of the presence of disulfide bonds), and ability to be synthesized, as well as selectivity in binding to target receptors or channels (Lewis and Garcia, 2003).

Studies on spiders of the Lycosidae family have shown that high levels of a small protein called Lycotoxin are found in the venom of these spiders (Consortium, 2019). According to the ArachnoServer spider venom database, the omega-Lycotoxins family is a P/Q-type VGCCs modulator (Pluzhnikov et al., 2007; Herzig et al., 2010). Although there are studies showing that omega-Lsp-1A can modulate P/Q-type VGCCs channels in the cerebellar purkinje cells and cornu ammonis 3 (CA_3_) region of the hippocampus (Fisyunov et al., 2005; Pluzhnikov et al., 2007). There has not been studied yet on the effects of omega-lycotoxin-Gsp2671e (OLG1e), extracted from *Lycosa praegrandis* venom, on spatial memory in excitotoxicity condition. In this condition, the OLG1e can modulate the over-active P/Q-VGCCs. Obviously, in the excitotoxicity process, in which over activity of the presynaptic calcium channels occurs, the OLG1e can potentially reduce subsequent damage and even their clinical manifestations such as forgetfulness. The aim of this study was to evaluate the effect of this state-dependent low molecular weight P/Q-type calcium modulator on the NMDA-induced memory deficits and synaptic quantification. To achieve this goal, *Lycosa praegrandis* venom extraction, determination of LD_50_ (for showing the toxicity of this crude venom), gel-filtration chromatography, capillary electrophoresis, and mass spectrometry were performed to purify and identify OLG1e small protein. Behavioral tests including Morris Water maze task and Novel Object recognition test were used to assess long and short-term memory. The SYN, SNAP-25, and SYT1 genes expression were measured *via* real time PCR for synaptic quantification. The field excitatory postsynaptic potentials (fEPSP) amplitude after LTP in Mossy Fiber pathway were recorded for evaluation of spatial memory and memory formation.

## Materials and methods

### Chemicals, reagents

All chemicals were purchased from Sigma Aldrich Company (Germany, Darmstadt) except for the others mentioned in the text.

### Spider collection and identification and venom extraction

For this study, specimens were collected alive from Iran (Zamani et al., 2021). The specimens were kept under suitable conditions, humidity (60%), and temperature (25^ο^C) and fed by crickets and mealworms. The specimens were identified using taxonomic keys (Nentwig et al., 2017).

Female spiders were separated to extract the venom. Specimens were anesthetized with CO_2_ in a small chamber, and the opisthosoma and carapace were removed under the stereomicroscope. Venom glands were collected into 4°C Phosphate buffered saline (PBS) (prepared in the laboratory with the following recipe: 137 mM NaCl, 3 mM KCl, 10 mM Na_2_PO_4_, 2 mM KH_2_PO_4_, and pH 7.4) and gently crushed with a glass stirrer for 30 min. Then, pieces of the venom gland were removed from the solution by centrifugation at 13000 rpm for 30 min at 4°C, and the supernatant was lyophilized and stored at -70°C. Protein concentration was measured by Bradford assay with bovine serum albumin as standard protein.

### Determination of lethal dose (LD50)

To determine the LD_50_, the albino mice (average weight 18–20 g) were intravenously IV) injected with the crude venom. For more accuracy in determining LD_50_, mice were selected instead of rats due to their higher sensitivity to the xenobiotics such as venom. After the injection, animals were followed up for 1 day. The Spearman-Karber method was used to calculate the LD_50_ dose for the crude venom and OLG1e Protein (Hamilton et al., 1977).

### Protein purification with gel-filtration chromatography

The lyophilized crude venom (10 mg) were resuspended in 1.5 ml of PBS buffer. The DNase (0.14 mg/ml) and RNase (0.14 mg/ml) enzymes were added to the sample and incubated for 2 h at 4°C. The clear solution was injected into a gel-filtration column (GE Healthcare HiLoad 16/600 Superdex^®^ 75 pg prep grade) and run over it using FPLC (Fast Protein Liquid Chromatography) system (Sykam, Germany). The column was washed with PBS Buffer. The injection volume was 1,200 μL and the flow rate was 0.7 ml/min. The fractions were observed with absorbance at 280 nm and collected in a 0.75 ml fraction. The chosen fractions marked on the graph were collected and injected to capillary electrophoresis.

### Protein purification and separation with capillary electrophoresis

Capillary electrophoresis test was performed by Agilent 7,100 equipped with a UV-Vis detector using a 50 μm uncoated silica column with a total length of 40 cm and a detector distance of 8.5 cm from the outlet. PBS buffer with pH 4.7 was used for both the sample, and running buffer. The capillary temperature was 25^ο^C and the sample was injected at 100 mBar for 5 s. Electrophoresis was performed for 5 min at 25 kV normal polarity. The marked peaks were collected, and protein concentration was determined by Bradford assay. The desired peak was re-injected into the device to ensure purity. The peaks obtained from this method were injected to HPLC-ESI-MS.

### Protein identification with mass spectrometry (HPLC-ESI-MS)

The high-performance liquid chromatography/electrospray ionization tandem mass spectrometry (HPLC-ESI-MS) analysis was performed by Waters Alliance 2695 HPLC-Micromass Quattro micro API Mass Spectrometer. Liquid chromatography separation was performed on Atlantis T3-C18 column (3µ, 2.1 × 100 mm) at 35°C. Mobile phases were 0.1% formic acid in acetonitrile and 0.1% formic acid in H_2_O. The gradient profile was 5% acetonitrile held for 0.2 min and linearly increased to 90% in 10 min. Then, it was held for 5 min, which decreased to 5% over 3 min and finally held for 4 min. The flow rate was 0.2 ml/min, and the injection volume was 5 μL. The mass spectrometry method was included in positive mode with the capillary voltage, which adjusted to 0.3 kV, and the source and dissolving temperatures were set at 120°C and 300°C, respectively, with a gas flow of200 L/h.

### Animals and experimental design

A total of 48 adult male Wistar rats, weighing 230–250 g were taken from the animal’s nest of the Faculty of Biological Sciences and Technology, University of Isfahan. They were kept in standard cages with controlled temperature (∼25°C) and humidity (∼40%), 12-h light; 12-h dark cycle, and free access to enough food and water. The study was approved by the ethics committee of the University of Isfahan.

The work was performed on male Wistar rats, which were accidentally spat into three groups (sixteen rats in each group). A small area on each rat’s skull was shaved while the head was fixed using a stereotaxic instrument (Stoelting Co., United States of America) to prepare for injection into the hippocampus. The PBS buffer was used as a vehicle for OLG1e and N- Methyl d-Aspartate (NMDA). Rats were assigned into the following groups:

Control Group: Received 2 µl of PBS in the CA3 sub-region of the hippocampus twice and with an interval of 15 min.

NMDA-treated Group: Received 2 µL of PBS followed by a single dose of NMDA (2µL, 5 μg/μl) in the CA3 sub-region of hippocampus 15 min later (Jarrard, 2002).

OLG1e -treated Group: Received 2 µL of OLG1e (1 μg/μL) in the CA3 sub-region of the hippocampus followed by a single dose of NMDA (2µL, 5 μg/μl) 15 min later (Hosseini-Sharifabad et al., 2021).

### Surgery and microinjection procedure

One week before the start of the behavioral tests, all the animals were deeply anesthetized with an intraperitoneal (i.p.) injection of phenobarbital (40 mg/kg). The phenobarbital was purchased from Martindale Pharma Company (Buckinghamshire, England). The animals were wrapped in towels and the eyes were covered with Vaseline during the operation. Then, the CA_3_ sub-region of the hippocampus (AP:−3.3 mm from bregma; ML: ±3 mm from midline; DV: 3.5 mm from the skull surface) was found using the Paxinos and Watson rat brain atlas (Paxinos and Watson, 2006). The injections were performed bilaterally on the hippocampi by stereotaxic apparatus.

The agents were bilaterally administered into the hippocampi using an injection needle (21 gauge) connected to a 5 µL Hamilton syringe through a polyethylene tube. The agents were slowly injected into the hippocampus area for 6 min. To avoid backflow of the fluid, the needle was slowly removed 2 min after the injection.

### Behavioral studies

The behavioral evaluations including Morris water maze and Novel object recognition tasks were performed a week after the stereotaxic surgery. These tests assessed the spatial and recognition memory, respectively. Prior to the behavioral tests, animals were acclimatized over 2 days in the laboratory area to get adjusted to the experimental condition and minimize stress.

### Morris water maze task

The Morris water maze test is one of the most common procedures to assess spatial memory and learning in rodents. The walls of the water maze room must have signs and symbols that rats can use their spatial memory to find the hidden platform in the target zone. Morris water maze task has three parts which are including habituation, training, and test. Rats were placed into different quadrants and trained to swim to reach a hidden platform for five consecutive days and four training courses per day. A trial terminated when the animal reached the platform during 90 s; otherwise, failed rat was gently guided toward the platform, and allowed to rest there for 30 s, and returned to the cage. The probe trial was performed 24 h after the last training session, the same as the training trial, except that the hidden platform was removed and each rat was allowed to search for it for 60 s. In the probe trial, each rat was released in the opposite quadrant of the platform facing the pool wall. All behavioral parameters of rat monitored by a video camera which was fixed to the ceiling above the center of the pool and connected to a computerized tracking system (Auto vision Software, Designed by BorjSanat Company, Tehran, Iran). (it has been completely described in previous work) (Ebrahimpour et al., 2018). In this task, the scape latency time of the training days, time spent and distance moved in the target quadrant, the entry into the target quadrant, velocity of rats, and swimming paths of rats during the probe trial were recorded to assess spatial memory and learning.

### Novel Object recognition task

The Novel Object recognition task is a procedure to assess recognition memory in rodents. This test is previously explained in a published study in details with few modification (Khani et al., 2018). In summary, its apparatus includes a cube arena (50-cm length×50-cm width×35-cm height, acrylic material), two different sets of objects including two massive aluminum cube and two massive aluminum triangle. Each object is available in triplicate. The objects are stable on the floor of arena. Novel Object recognition task has three phases, including habituation, training, and test. Animals are acclimated in the empty test box for 5 min, after which they are exposed to objects during two trials, each lasting for 5 min. Animals are presented with two identical objects (A_1_ and A_2_) to explore for a training phase. After 60 min, following the removal of the objects, the rats are again presented with two objects, one of which is from the initial exposure A) and the other is novel B) for that phase. In this test, d_2_ (an index which indicates the discrimination between the new and the familiar objects) and R (an index which indicates the recognition between the new and the familiar objects based on the exploration time spent close to the new object from the total exploration time e)) were calculated to evaluate the recognition memory.

$$d2=\frac{\left(eB-eA\right)}{\left(eB+eA\right)}\;R=\frac{eB}{\left(eB+eA\right)}$$

### RNA extraction, complementary DNA (cDNA) synthesis and quantitative Real-Time PCR

The mRNA extraction executed as formerly described by Beheshti et al. (2020) and mRNA expression measurement was performed for the three groups (Beheshti et al., 2020). Total RNA was extracted from the hippocampus samples using RNX-PLUS reagent (SinaClon, Iran). cDNA was synthesized according to the manufacturer’s protocol with a cDNA synthesis kit (Takara, Japan). Real-time PCR assay was performed by StepOnePlus™ Real-Time PCR System. The sequences of Real-time PCR primers were as follows: 5′- AGG​GCC​TAT​GAT​GGA​CTT​TCT​G-3′, and 5′-TCC​GTG​GCC​ATC​TTC​ACA​TC-3′ for ‌‌mice Synaptophysin, 5′-CCT​CCA​CTC​TTG​CTA​CCT​GC-3′, R-5′-TCCTCTGCATCTCCTCCAGT-3′) for ‌‌mice SNAP-25, 5′-CGG​CAA​ACT​GAC​TGT​CAT​TC-3′ and 5′-GCC CCA GTG CTG TTG TAA CCA-3′ for ‌‌mice Synaptotagmin 1, and 5′-CAG​GGC​TGC​CTT​CTC​TTG​TG-3′, and 5′-GAT​GGT​GAT​GGG​TTT​CCC​GT-3′ for mice Glyceraldehyde-3-phosphate dehydrogenase (GAPDH). Relative genomic expression was calculated by the 2^−ΔΔCT^ method. The mRNA levels were normalized to that of GAPDH (Beheshti et al., 2020).

### Cresyl violet staining

Tissue sagittal slides were deparaffinized 2 times in xylene, each for 10 min**.** Rehydration was performed using gradient alcohol (100%, 95%, 70%, and 50% alcohol) each time for 5 min. The brain slides were stained in cresyl violet solution (0.25% cresyl violet, 0.8% glacial acetic acid, 0.6 mM sodium acetate) for 20 min. The dehydration was done using gradient alcohol (70%, 95%, and 100% alcohol) each time for 3 min. Tissue slides were visualized using a BX40 Olympus light microscopy.

### Electrophysiological study

The rats (six in each group) were anesthetized with urethane (1.5 g/kg; intraperitoneal injection) dissolved in 0.9% normal saline solution. The surgery and LTP recording procedures from the hippocampal CA_3_ area were executed as formerly described by Ramos-Languren Escobar, (2013) (Ramos-Languren and Escobar, 2013). In brief, the skull was exposed and two small holes were drilled at the positions of the stimulating and recording electrodes. A concentric bipolar stimulating electrode (stainless steel, 0.125 mm diameter, Advent, United Kingdom) was placed in the Mossy Fiber (AP:−3.6 mm from bregma; ML: ±2.3 mm from midline; DV: 3.5 mm from the skull surface). A unipolar stainless steel recording electrode was lowered into the CA_3_ area (AP: −3.3 mm from bregma; ML: ±3 mm from midline; DV: 3.3–3.7 mm from the skull surface) until the maximal response was observed. Baseline were recorded 30 min before and 60 min after high-frequency stimulation (HFS). The LTP was induced using HFS protocols at a100 Hz (4 bursts of 50 stimuli, 0.15 m archive stimulus duration). . The fEPSP response passed through an analog-to-digital interface (Data acquisition ScienceBeam-D3111), signals were transmitted to a computer and the data were analyzed using custom software applications (eProbe software).

### Data analysis

Statistical analysis was performed using Graph Pad Prism statistics software (version 8.4.3). Statistical data were evaluated by the D'Agostino-Pearson omnibus test to examine the normal distribution. The data collected were analyzed by one-way, one-way repeated measures, and two-way analysis of variance (ANOVA). For multiple comparisons, Tukey’s test was used. All data were shown as the mean ± standard errors of the means (*p* < 0.05 was considered a significant difference).

## Results

### Spider collection, identification and venom extraction

Fifty-five spiders were collected from nature, and separated by species, and gender. Seventeen female specimens of *Lycosa praegrandis* were picked out for the next phase of the study.

150 mg of the lyophilized crude venom was extracted from seventeen specimens. Then, 10 mg of the crude venom was dissolved in PBS buffer. The protein concentration of the lyophilized crude venom was 0.65 mg/ml, which determined by the Bradford method.

### Determination of LD50

Based on IV injection of the crude venom and OLG1e Protein into the albino mice, LD_50_ were determined as 37.35 mg/kg and not toxic respectively.

### Protein purification with gel-filtration chromatography

**FIGURE 1:**
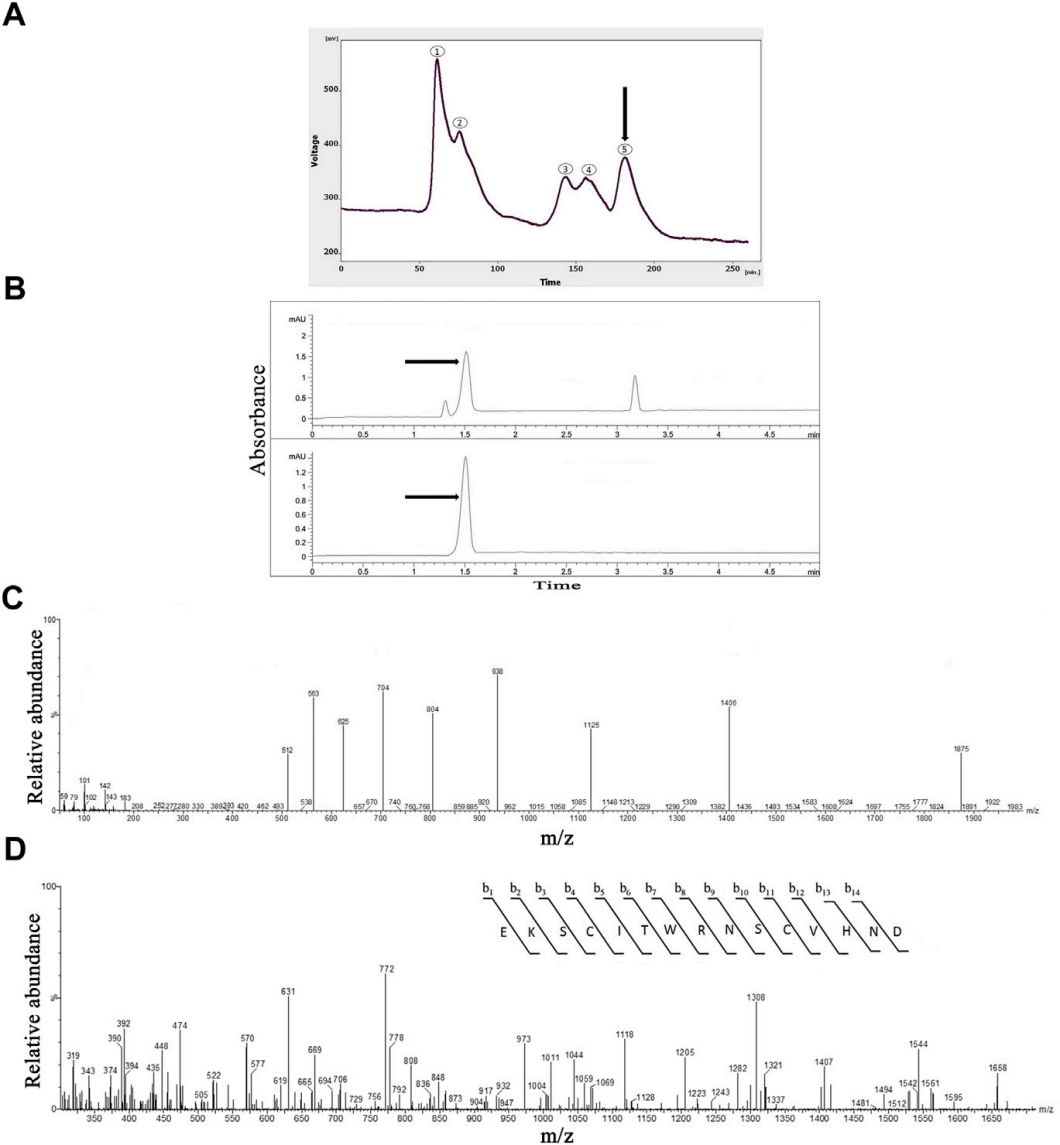
The Purification, and identification of OLG1e from the *Lycosa praegrandis* crude venom. **(A)** Gel-filtration chromatography of the crude venom performed on a GE Healthcare HiLoad 16/600 Superdex^®^ 75 pg prep grade column in 1 M PBS (pH 7.4), flow rate 0.7 ml/min. The fifth fraction is shown by black arrow. **(B)** Capillary electrophoresis of the sixth fraction was performed on a 50 μm uncoated silica column 1 M PBS (pH 4.7) for 5 min. The OLG1e fraction is shown by black arrow. **(C)** HPLC-ESI-MS of the OLG1e fraction was performed on a of Atlantis T3-C18 column. The spectrum results are shown in part **(C)**.**(D)** N-terminal partial sequencing of the OLG1e protein. The singly charged ion of the N-terminal peptide (m/z, 1,406) was subjected to the fragmentation in the ion trap mass analyzer. Fragment ions observed are indicated above and below the peptide sequence.

The gel-filtration chromatography of the crude venom provided five peaks (Figure 1A). The fifth fraction was collected from 175 to 200 min. Totally, 1.47 mg of the lyophilized fifth fraction was obtained from 17.5 ml of solution taken from FPLC. The protein concentration of the fifth fraction of the crude venom was 1.08 mg/ml which determined by the Bradford method.

### Protein purification with capillary electrophoresis (CE)

The selected fraction had three peaks in CE technique at 1.3, 1.5, and 3.18 min. This graph was rendered three narrow peaks. The second peak project to HPLC-ESI-MS for mass determination. The pI was determined for the second peak 8.58 (Figure 1B).

### Protein identification with mass spectrometry (HPLC-ESI-MS)

According to the ArachnoServer spider venom database (Herzig et al., 2010), the molecular mass of OLG1e was determined 5,599 Da. The results of the spectrum indicated the presence of this bio-active small protein. The mass-to-charge ratio of this state-dependent low molecular weight P/Q-type calcium modulator was quite consistent with OLG1e was determined 5,599 Da. The charge-to-mass ratio of OLG1e is as follows: (M + Na+2H)^3+^ = 1874.6 (M + Na+3H)^4+^ = 1,406.25, (M + Na+4H)^5+^ = 1,125.2 (Figure 1C). Mass/Mass spectrum of quadro charge ion at 1,406.25 was selected for N-terminal partial sequencing as follows: b_3_ = 345, b_4_ = 448, b_5_ = 561, b_6_ = 662, b_7_ = 848_,_ b_8_ = 1004_,_ b_9_ = 1118_,_ b_10_ = 1205_,_ b_11_ = 1308_,_ b_12_ = 1407_,_ b_13_ = 1544_,_ b_14_ = 1,658. The results of the spectrum indicated the consistence with OLG1e (Accession number: A9XDG5) sequence (Figure 1D).

### Morris Water maze task

**FIGURE 2:**
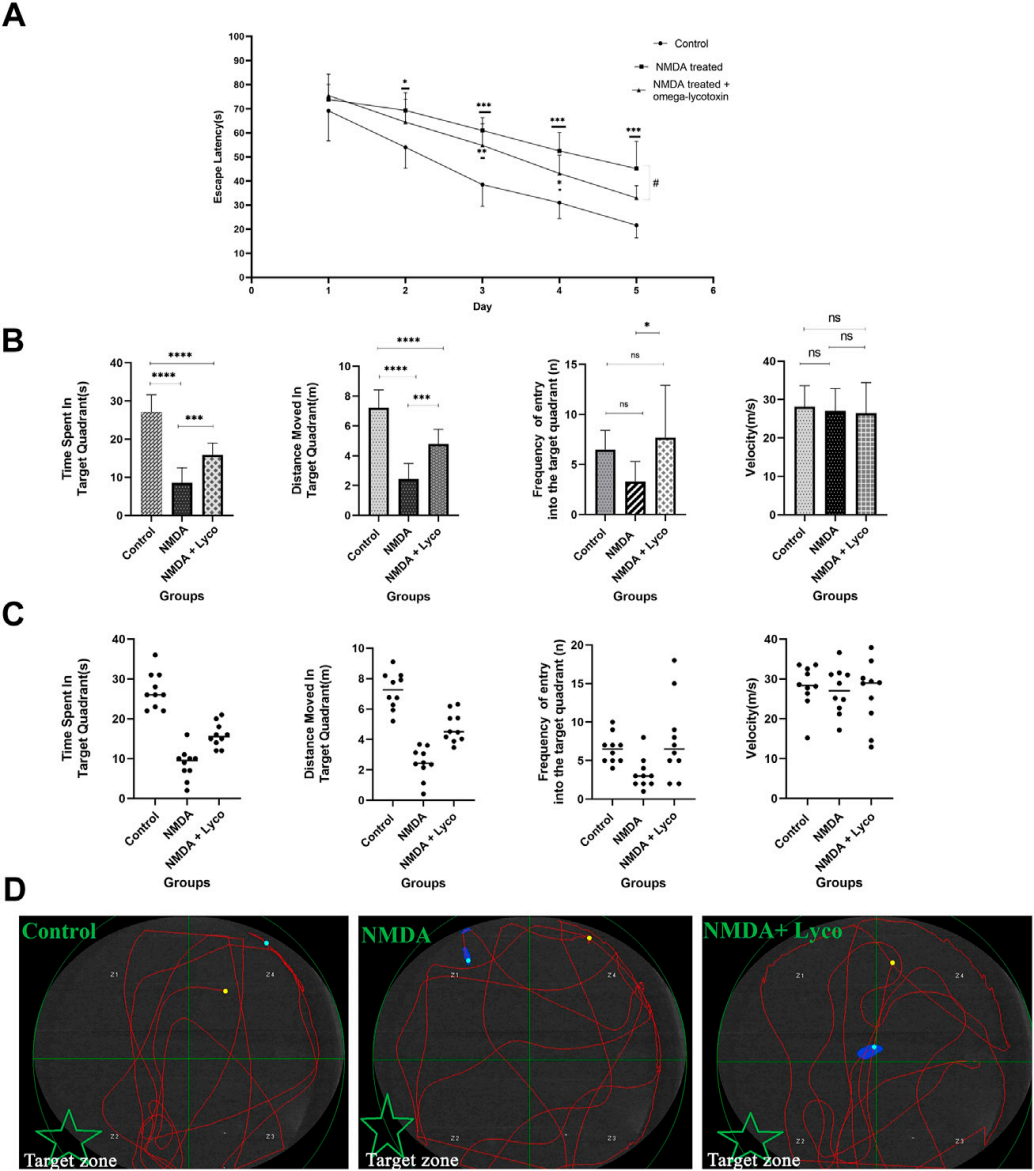
The Morris water maze test **(A)** The effect of treatment with OLG1e on the average escape latency time during the training days. The data are shown as means ± SEM of 10 rats per group. (*p < 0.05 and **p < 0.01 and ***p < 0.001 and ****p < 0.0001) and (#*p* < 0.05). **(B)** The effect of treatment with OLG1e on time spent in the target quadrant, distance moved in the target quadrant, frequency of entry into the target quadrant from the test day. The data are shown as means ± SEM of 10 rats per group (*p < 0.05 and **p < 0.01 and ***p < 0.001 and ****p < 0.0001). **(C)** Scatter plot of part **(B) (D)** The swim path traces from the test day. The target zone is indicated with green star.

As shown in Figure 2A, NMDA-treated with a single dose of OLG1e decreased the escape latency time compared to NMDA-treatment group on day 5 (*p* < 0.05). The intra-hippocampal administration of NMDA significantly decreased the time spent (*p* < 0.0001), and distance moved in the target quadrant (*p* < 0.0001) compared to the control group. NMDA-treatment with administration of OLG1e increased the time spent (*p* < 0.001), distance moved in the target quadrant (*p* < 0.001), and frequency of the entry into the target quadrant of the maze compered to NMDA-treatment group. Also, significant differences in the time spent (*p* < 0.0001), and distance moved (*p* < 0.0001) in the target quadrant of the maze were observed between the control, and NMDA-treated + Lyco group. No statistical differences were observed between the experimental groups regarding the entry into the target quadrant and velocity of rats swim except mentioned one (Figure 2B, C).

In addition, as presented in Figure 2, part ِ, the swimming path of the NMDA-treated rats was irregular and did not have a specific pattern, while the path of control rats was regular round movement into the target area. In spite of the NMDA-treated rats, the swimming path of the NMDA-treated + Lyco group was regular, and similar to the control group.

### Novel Object recognition task

**FIGURE 3:**
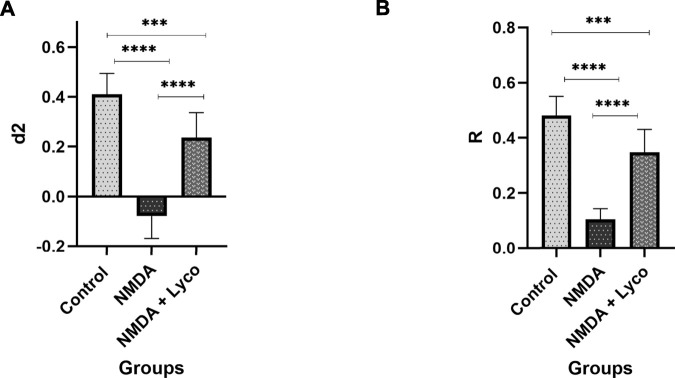
The Novel object recognition test **(A)** The effect of treatment with OLG1e on the d_2_ index. The data are shown as means ± SEM of 10 rats per group. (*p < 0.05 and **p < 0.01 and ***p < 0.001 and ****p < 0.0001) **(B)** The effect of treatment with OLG1e on the R index. The data are shown as means ± SEM of 10 rats per group (*p < 0.05 and **p < 0.01 and ***p < 0.001 and ****p < 0.0001).

According to Figure 3, administration of a single dose of NMDA decreased the d_2_ (*p* < 0.0001) and R (*p* < 0.0001) indexes compared to the control group. Administration of OLG1e followed by NMDA injection, increased the d_2_ (*p* < 0.0001) and R (*p* < 0.0001) indexes compared to NMDA-treated group. Significant differences were observed between the two examined groups (NMDA-treated, and NMDA-treated with a single dose of OLG1e) for d_2_ (*p* < 0.001) and R (*p* < 0.001) indexes in the Novel Object recognition test.

### Quantitative Real-Time PCR

**FIGURE 4:**
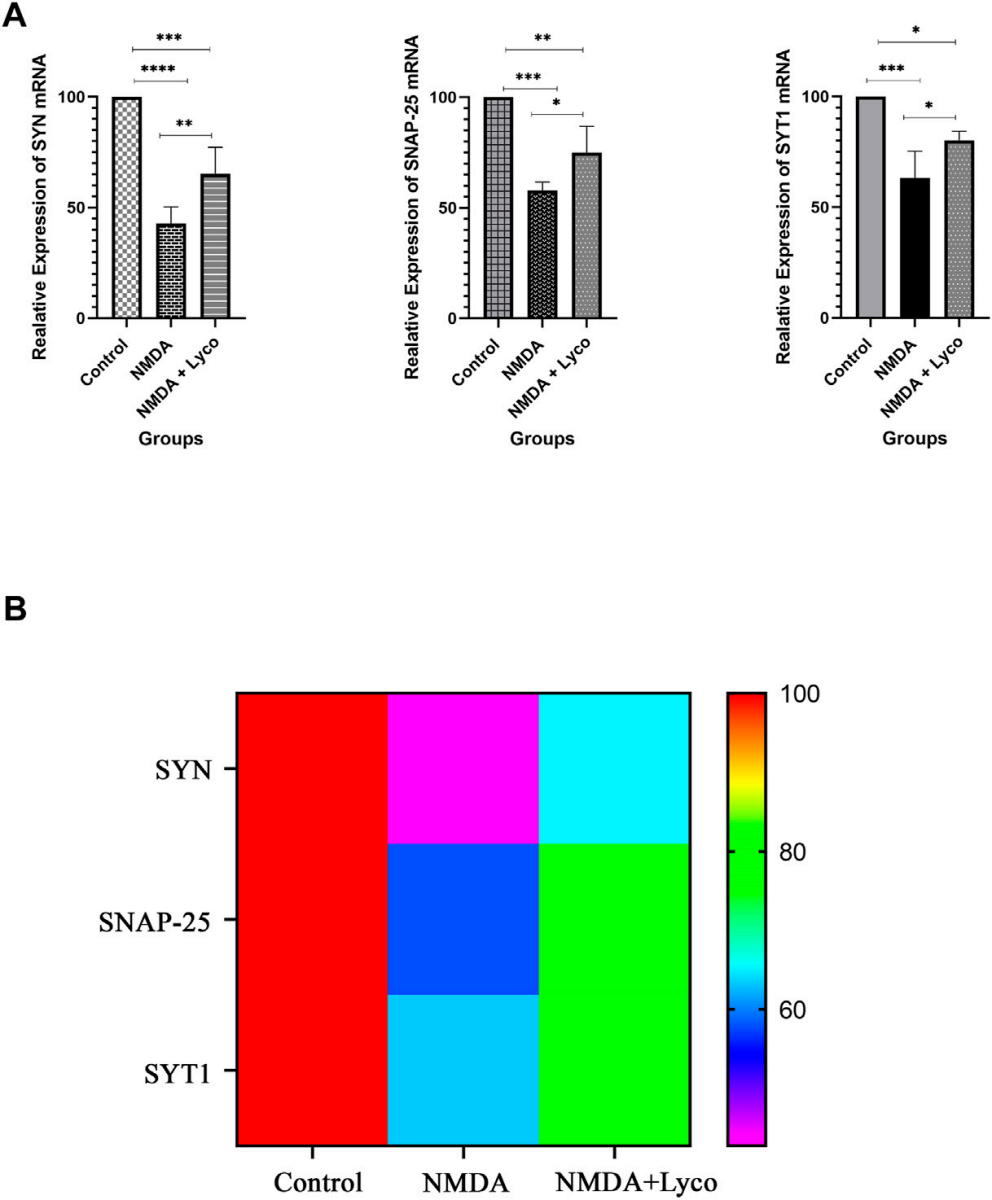
The Quantitative Real-Time PCR **(A)** The effect of treatment with OLG1e on the hippocampal SYN, SNAP-25, and SYT1 mRNA levels. The data are shown as means ± SEM of six rats per group. (*p < 0.05 and **p < 0.01 and ***p < 0.001 and ****p < 0.0001) **(B)** Heat map for SYN, SNAP-25, and SYT1 mRNA levels (Two-way ANOVA).

Figure 4 displays the effect of NMDA-treatment and NMDA-treated + lyco groups on SYN, SNAP-25, and SYT1 genes expression in the rat hippocampus. This figure revealed that administration of NMDA in the rat hippocampus distinctly decreased SYN (*p* < 0.0001), SNAP-25 (*p* < 0.001), and SYT1 (*p* < 0.001) mRNAs expression when compared to the control group. The NMDA-treated group with a single dosage injection of OLG1e obviously increased SYN (*p* < 0.01), SNAP-25 (*p* < 0.05), and SYT1 (*p* < 0.05) mRNAs expression with respect to the NMDA-treated group. Significant differences were observed between the two examined groups for SYN (*p* < 0.001), SNAP-25 (*p* < 0.01), and SYT1 (*p* < 0.05) mRNAs expression: NMDA-treated, and NMDA-treated with a single dose of OLG1e.

### Cresyl violet staining

**FIGURE 5:**
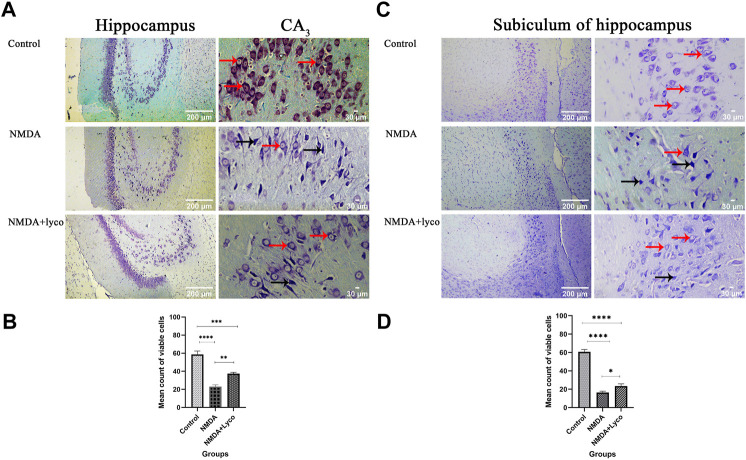
The Cresyl Violet Staining of rat hippocampus. **(A)** The healthy (Narrow red arrow) and dead (Narrow black arrow) pyramidal neurons are indicate in the CA_3_ area. **(B)** The effect of treatment with OLG1e on the mean count of viable cells in CA3 of the hippocampus. The data are shown as means ± SEM of three rats per group (*p < 0.05 and **p < 0.01 and ***p < 0.001 and ****p < 0.0001). **(C)** The healthy (Narrow red arrow) and dead (Narrow black arrow) pyramidal neurons are indicate in the subiculum area. **(D)** The effect of treatment with OLG1e on the mean count of viable cells in subiculum of the hippocampus. The data are shown as means ± SEM of three rats per group (*p < 0.05 and **p < 0.01 and ***p < 0.001 and ****p < 0.0001).

Our data showed that the single injection of NMDA, significantly decreased the mean of viable cells count in CA_3_ region (*p* < 0.0001), and subiculum (*p* < 0.0001) of the hippocampus compared to the control group. The single administration of OLG1e with injection of NMDA, distinctly enhanced the mean count of viable cells in CA_3_ region (*p* < 0.01) and subiculum (*p* < 0.05) of the hippocampus with respect to the NMDA-treated group. The NMDA-treated + Lyco group showed a significant difference in CA_3_ region (*p* < 0.001) and subiculum (*p* < 0.0001) of the hippocampus compared to the control group (Figure 5).

### Mossy fiber circuit LTP

**FIGURE 6:**
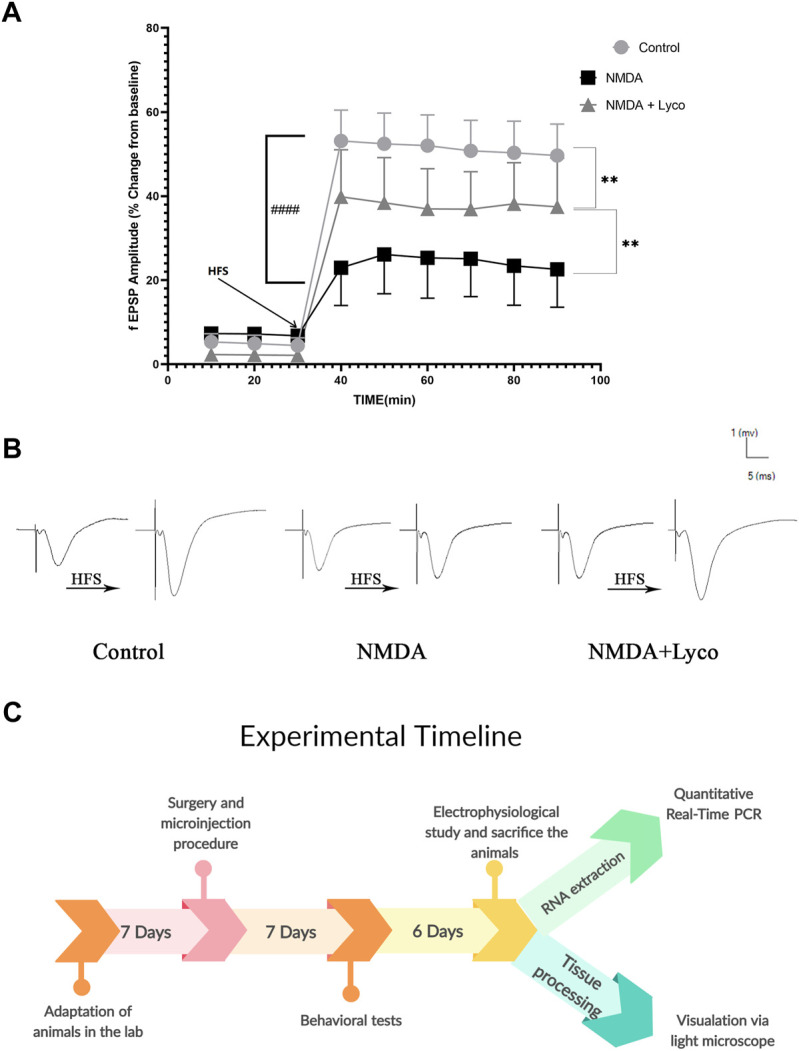
Mossy Fiber circuit LTP **(A)** Long-term potentiation (LTP) curves of the field excitatory postsynaptic potential (fEPSP) amplitude in the hippocampal CA_3_ for the all groups (*n* = 6). The data are shown as means ± SEM of six rats per group. (*p < 0.05 and **p < 0.01 and ***p < 0.001 and ****p < 0.0001), and (^####^ *p* < 0.0001). **(B)** Sample traces of typical recorded fEPSPs in the hippocampal CA3 neurons before and after high-frequency stimulation (HFS) induction for the long-term potentiation (LTP) in experimental groups. **(C)** Experimental timeline. Timeline showing the sequence of events and behavioral testing the animals underwent.

According to Figure 6A, the injection of NMDA significantly decreased the field excitatory postsynaptic potentials (fEPSP) amplitude in the NMDA-treated group after LTP induction in the CA3 of the hippocampus when compared to the control group (*p* < 0.0001). The administration of a single dose of OLG1e after the injection of NMDA in the NMDA-treated + Lyco group remarkably increased the fEPSP amplitude after LTP induction with respect to the NMDA-treated group (*p <* 0.01). The fEPSP amplitude in the NMDA-treated + Lyco group had a significant difference compared to the control group (*p* < 0.01).

Figure 6B, demonstrates the traces of the recorded fEPSPs in the hippocampal CA_3_ neurons, before and after LTP induction using the high-frequency stimulation (HFS) technique in all the compared groups.

The experimental timeline showed the sequence of the procedures of the experiments (Figure 6C).

## Discussion

The *Lycosa praegrandis* species is a member of lycosidae family. This spider is a nocturnal runner. The *Lycosa singoriensis* species is a sister group for *Lycosa praegrandis* (*Jocqué and Alderweireldt, 2005*; *Wheeler et al., 2017*; *Zamani et al., 2021*)*.* Based on Uniprot database, lycosidae family members have lycotoxins. There are three types of lycotoxins including: omega, U, and M. Omega-lycotoxin types have a modulatory or blocker effect on P/Q-type VGCCs (Consortium, 2019). Therefore, the *Lycosa praegrandis* is expected to have a lycotoxin.

In the present study, the *Lycosa praegrandis* crude venom was investigated and a P/Q–type VGCC was purified (OLG1e). In addition, the effect of OLG1e on memory impairment was explored using a rat model of NMDA-induced excitotoxicity. This state-dependent small bio-active protein was purified through a unique method in which PBS buffer and low-pressure in the column were used for the purification process. It is different compared to some previous studies, in which acetonitrile, methanol, and high pressure method such as high-performance liquid chromatography (HPLC) have been used (Fontana et al., 2003; Vassilevski et al., 2010; Xiao et al., 2018; Sivaramakrishnan et al., 2022). Consequently, the protein had post-translational modifications and was in its active and native structure. Folding and post-translational modifications of native structure of the protein are very sensitive and could be changed easily (Müller, 2018). Therefore, for purification of the protein, it is necessary to use an appropriate solvent, and a low-pressure method such as gel-filtration chromatography and CE.

The concentration and amount of *Lycosa praegrandis* venom proteins, obtained in the current study, had similar levels of proteins as previously was studied (Liu et al., 2009). Considering the low LD_50_ amount of this crude venom, the injection of this venom was safe in rats. Meanwhile, the LD_50_ amount of purified fraction is far less than the LD_50_ amount of crude venom. It is because the harmful digestive enzymes such as hyaluronidase and chitinase have high molecular weights (Ozegowski et al., 1994; Hamid et al., 2013), despite the used fraction which had a low molecular weight. The gel-filtration pattern of *Lycosa praegrandis* had five peaks. The proteins in the fifth fraction had the lowest mass among the others. The last fraction was injected to CE. The second peak was collected and reinjected to ensure the purity of this small protein. This action confirmed the purity of this peak as well as the accuracy of the collected peaks. The value of this peak was also significantly higher than the other peaks. This peak was selected to continue the study and injected into the HPLC-ESI-MS device. The resulted spectrum of this small protein was consist with OLG1e. According to the Uniprot database, the OLG1e is potentially an active modulator for P/Q–type VGCCs (Consortium, 2019).

P/Q–type VGCCs are presynaptic. These channels have a key role for the release of neurotransmitters, and signal transduction (Nimmrich and Gross, 2012). The chief neurotransmitter in the pyramidal cells of the hippocampus is glutamate. Glutamate has a vital role in memory formation (Dobrek and Thor, 2011). Excessive amounts of this neurotransmitter is destructive for neuronal cells (Lau and Tymianski, 2010). Therefore, the balance of glutamate level is crucial for synaptic plasticity and neuronal survival (Bin Ibrahim et al., 2022; Hayashi, 2022). In AD, this balance is disappeared, which ends to induction of glutamate-induced excitotoxicity (Di et al., 2010). During this condition, the pyramidal cells would be eliminated due to generation of various free radicals, mitochondrial dysfunction, and induction of apoptosis and necrosis factors (Lai et al., 2014; Verma et al., 2022). This process can be reduced with an effective P/Q–type VGCCs modulator (Schurr, 2004; Nimmrich and Gross, 2012). Based on some previous studies on excitotoxicity models, hyper-stimulation of glutamate receptors is a result of the administration of NMDA in the rat hippocampus (Jarrard, 2002; Gilmour et al., 2012; Farzamfard et al., 2022; Morland and Nordengen, 2022).

Our data were in line with previous studies, showing that glutamate-induced excitotoxicity resulted in memory performance elimination (Farzamfard et al., 2022; Parfait et al., 2022). In Morris Water Maze Task, rats use the clues on the wall to find the hidden platform under the water. This process is memorized by the hippocampus. Performance of the CA_3_ sub-region and subiculum of the hippocampus is necessary to complete this task. Pyramidal cells of the CA_3_ are highly connected together, and have a self-excitation characteristic (Morgado Bernal and Segura Torres, 2022). Therefore, these cells can excite each other and amplify the excitotoxicity effect of NMDA administration. Also, LTP in Mossy fiber on CA_3_ interneurons, and pyramidal cells is largely NMDA-independent and these neurons have large presynaptic terminals and frequent release sites (Alkadhi, 2021). These features make CA_3_ a good area for demonstration of the impact of P/Q–type VGCCs modulators. Moreover, subiculum has a key role in spatial memory and navigation (Matsumoto et al., 2019). In the current study, the NMDA-treated rats failed to complete these tasks. This impairment was induced by the hyper-stimulation of NMDARs (Dobrek and Thor, 2011; Errico et al., 2011), and reduced when the P/Q–type VGCCs modulator was used (Hosseini-Sharifabad et al., 2021). This amelioration depends on the regulation of glutamate release by modulating P/Q–type VGCCs.

Our data showed that intra-hippocampal injection of NMDA reduces fEPSP after LTP induction due to the destructive effect of excitotoxicity. However, administration of OLG1e, restored the fEPSP to lower levels than normal by modulation of P/Q–type VGCCs and prevention of excitotoxicity induction.

Regarding the crucial roles of SYN, SNAP-25, and SYT1 in synapse firing and LTP, these findings show that the NMDA-induced neurotoxicity and memory defects occurred due to the decrease in SYN, SNAP-25, and SYT1 mRNAs expression (Li et al., 2012; You et al., 2018). OLG1e can reverse this destructive effect and improve memory and learning. Moreover, SYN, SNAP-25, and SYT1 have an undeniable task in the LTP process. Excitatory neuron firing, release and uptake of glutamate, and Synaptic activity provide a cellular basis for learning and particularly memory in the hippocampus (Tampellini et al., 2010; Xu et al., 2019). Consequently, SYN, SNAP-25, and SYT1 as presynaptic proteins with calcium binding property, have a dominant role in the release of glutamate, fusion of synaptic vesicles, and synaptic plasticity (Valtorta et al., 2004; Tampellini et al., 2010; Zhang et al., 2014; Li and Kavalali, 2017). Decrease in the rate or absence of SYN, SNAP-25, and SYT1 mRNAs ends to learning and memory impairment. Therefore, any reduction or loss of SYN, SNAP-25, and SYT1 mRNAs due to glutamate-induced excitotoxicity can potentially reduce these markers. Anyway, this data shows that a single dosage administration of NMDA, strikingly reduces the SYN, SNAP-25, and SYT1 mRNAs expression in the rat hippocampus. Meanwhile, NMDA-treatment with a single dose injection of OLG1e can restore this reduction to an acceptable level by modulating of P/Q–type VGCCs. Also, our data revealed that the expression of SNAP-25, and SYT1 mRNAs is more than SYN mRNA. SNAP-25 and SYT1 are expressed in postsynaptic neurons as well as presynaptic terminals (Antonucci et al., 2016; Hussain et al., 2017). When the measurement of the hippocampus mRNAs were performed using quantitative Real-Time PCR, their mRNAs were evaluated in both pre and post synaptic terminals.

The hippocampal section of control group showed a usual pattern. The pyramidal cells and their nucleus had a normal shape, size, and membrane. Despite this, the hippocampal section of the NMDA-treated and NMDA-treated with a single intra-hippocampus administration of OLG1e rats displayed elimination of CA_3_ and subiculum pyramidal cells of the hippocampus. Moreover, these hippocampal tissues represented the fragmented nuclei, and membrane budding. The NMDA-treated and NMDA-treated + lyco sections can be discriminated by abnormal and normal pyramidal cells in CA_3_ and subiculum, respectively. The dorsal sections of the hippocampus were investigated in the current study. This section of the hippocampus has a bold role in spatial memory processing, which is confirmed by the Morris water maze results (Fanselow and Dong, 2010; Lee et al., 2019).

In conclusion the induced excitotoxicity by NMDA injection into the rat hippocampus, led to death of CA_3_ pyramidal neurons, decrease fEPSP after LTP induction, and down-regulation of SYN, SNAP-25, and SYT1 mRNAs. In contrast, the administration of OLG1e as P/Q–type VGCCs in NMDA-treated rats led to inhibition of neuronal cell death and prevention of pyramidal cell elimination, due to modulation of P/Q–type VGCCs, as well as the increase in the amount of SYN, SNAP-25, and SYT1 mRNAs and restored the fEPSP to upper levels than NMDA-treated group. The results indicate that purified OLG1e as a P/Q-type VGCC modulator has an ameliorative effect on excitotoxicity-induced memory defects and prevents the impairment of pyramidal neurons in the rat hippocampus.

The results of the current study revealed that the purified OLG1e can affect the P/Q–type VGCCs in the excitotoxicity condition and improve the memory performance. The importance of this increases when the relationship between Alzheimer’s disease and channelopathy is taken into account. This study can be considered as a start point for next evaluations of the effect of OLG1e on the cognitive abilities such as; pain sensitivity, associative learning, sensorimotor and locomotor ability. In addition, evaluation of the effect of bio-active small proteins on the N-type VGCCs can be considered for the future researches.

## Data Availability

The original contributions presented in the study are included in the article and further inquiries can be directed to the corresponding authors.
